# Triple Antithrombotic Therapy (Triple Therapy) After Percutaneous Coronary Intervention in Chronic Anticoagulation: A Literature Review

**DOI:** 10.7759/cureus.21810

**Published:** 2022-02-01

**Authors:** Ali Hussain, Amjad Minhas, Usman Sarwar, Hassan Tahir

**Affiliations:** 1 Internal Medicine, Sentara Albemarle Medical Center, Elizabeth City, USA; 2 Medicine, Sentara Obici Hospital, Suffolk, USA; 3 Cardiology, University of South Alabama, Mobile, USA; 4 Internal Medicine, University of South Alabama, Mobile, USA; 5 Cardiology, Lake Cumberland Regional Hospital, Somerset, USA

**Keywords:** non-valvular atrial fibrillation, dual-antiplatelet therapy (dapt), atrial fibrillation and pci, cad: coronary artery disease, therapeutic anticoagulation

## Abstract

Selecting anticoagulation therapy for patients with atrial fibrillation and coronary artery disease has always been challenging for physicians. The treatment modalities have evolved with time. Oral anticoagulation with warfarin was used in the initial era of stenting to prevent stent thrombosis, and dual antiplatelet therapy is the current recommendation. Triple anticoagulation therapy, i.e., aspirin, P2Y12 inhibitor, and oral anticoagulation, is associated with higher bleeding episodes and mortality compared to the combination of an anticoagulant and a P2Y12 inhibitor.

## Introduction and background

In patients who need oral anticoagulation and antiplatelet therapy, it is clinically challenging to balance the benefit and risks involved with aggressive antithrombotic treatment. Atrial fibrillation (AF) accounts for 20% to 30% of ischemic strokes in the United States [1]. Despite guideline-directed medical therapy, a recently published study showed a high prevalence of coronary artery disease (CAD) in symptomatic AF [2]. Optimum treatment preferences for AF patients who undergo percutaneous coronary intervention (PCI) have been controversial. Dual antiplatelet therapy (DAPT), i.e., aspirin plus P2Y12 inhibitors, is used for secondary prevention of coronary events and protection against stent thrombosis but does not provide complete protection against stroke in AF [3-5]. Two of the significant indications of oral anticoagulation, in those receiving direct-acting oral anticoagulants (DOACs) and vitamin K antagonist (VKA), are AF and venous thromboembolism (VTE). DAPT has not been indicated for either AF or VTE. Ten percent of patients who undergo PCI also have AF, and others have VTE [6]. Thus, anticoagulation is imperative in these patients. It becomes challenging for these patients to choose the proper antithrombotic regimen. Combining DAPT and oral anticoagulants (OACs), also called triple anticoagulation or triple therapy, significantly increases all-cause hospitalization (adjusted hazard ratio [HR] 1.75; 95% confidence interval [CI], 1.35-2.26; P < 0.0001) [7]. DOAC-based double therapy decreases the bleeding risk compared with triple therapy (risk ratio [RR] 0.66, 95% CI 0.56-0.78; P < 0.0001; I^2^ = 69%), for all bleeding definitions. There were no major differences in all-cause and cardiovascular mortality, stroke, and major adverse events [8].

## Review

The management of AF after PCI is a common challenge in everyday practice. Before 2016, it was common to use triple therapy post-PCI in patients with AF or VTE. Before we go into the details of different clinical trials, we should assess the individual patient risk for bleeding and thrombosis. The most important aspect is the optimum time to stop aspirin (acetylsalicylic acid [ASA]) or P2Y12 inhibitor and the potential thrombotic risks associated with continuation. The other aspect is the selection of DOACs versus VKA. Table 1 describes major trials comparing efficacies of antithrombotic therapy.

**Table 1:** Major trials comparing efficacies of antithrombotic therapy post-discharge from hospitals OAC: oral anticoagulant, ASA: acetylsalicylic acid (aspirin), DES: drug-eluting stent, MI: myocardial infarction, TIMI: thrombolysis in myocardial infarction, VKA: vitamin K antagonist, INR: international normalized ratio, AF: atrial fibrillation, G: group, DAPT: dual antiplatelet therapy, CAD: coronary artery disease, CV: cardiovascular, ACS: acute coronary syndrome, CRNM: clinically relevant non-major *Apixaban 2.5 mg twice daily used if at least 80 years of age, weighed < 60 kg, or with a creatinine level of at least 1.5 mg/dl **Edoxaban 30 mg/day used if one or more of the following factors were present: creatinine clearance 15-50 ml/min, bodyweight ≤60 kg, or concomitant use of specified potent P-glycoprotein inhibitors.

Trial/year	Population	Primary endpoint	Medication regimen	Findings	Conclusion
WOEST/2013 [9]	573 patients on OAC undergoing PCI assigned to oral clopidogrel alone or a combination of ASA and clopidogrel	Bleeding episode within 1 year of PCI	OAC with clopidogrel 75 mg/day (double therapy) OAC with clopidogrel 75 mg/day plus ASA 80-100 mg/day (triple therapy)	In the double therapy group, 19.4% had bleeding episodes compared to 44.4% in the triple therapy group.	The use of clopidogrel without ASA was associated with a significant reduction in bleeding episodes without increased thrombotic events.
ISAR-TRIPLE/2015 [10]	614 patients receiving OAC underwent DES implantation for stable angina or ACS.	Death, MI, definite stent thrombosis, stroke, or TIMI major bleeding at 9 months	Clopidogrel 75 mg/day for 6 weeks or 6 months; aspirin 75-200 mg/day; and a VKA	The primary endpoint occurred in 9.8% in the 6-week group compared to 8.8% in the 6-month group. No major differences in ischemic events were reported.	6-week triple therapy was not superior to 6-month triple therapy concerning clinical outcomes.
PIONEER AF-PCI/2016 [11]	2124 AF patients with PCI and stenting were divided into 3 groups with a 1:1:1 ratio	Clinically significant bleeding	G1: low-dose rivaroxaban (15 mg/day) and P2Y12 inhibitor for 12 months; G2: very-low-dose rivaroxaban (2.5 mg twice daily) and DAPT for 1, 6, or 12 months; G3: VKA and DAPT for 1, 6, or 12 months	Clinically significant bleeding rates were 16.8%, 18%, and 26.7% in G1, G2, and G3, respectively. Death rates from CV causes, MI, or strokes were 6.5%, 5.6%, and 6% for G1, G2, and G3, respectively.	Low-dose rivaroxaban plus P2Y12 inhibitor for 12 months or very-low-dose rivaroxaban + DAPT for 1, 6, or 12 months showed lower rates of clinically significant bleeding than VKA + DAPT for 1, 6, or 12 months.
RE-DUAL PCI/2017 [12]	2725 patients with AF and PCI	Thromboembolic events, death, or unplanned revascularization	Triple therapy (warfarin plus a P2Y12 inhibitor and aspirin) for 1-3 months; dual therapy (dabigatran plus a P2Y12 inhibitor and no aspirin)	The primary endpoint was 15.4% in the dabigatran 110-mg dual therapy group vs. 26.9% in the triple therapy group and 20.2% in the dabigatran 150-mg dual therapy group vs. 25.7% in the corresponding triple therapy group.	The bleeding risk was low among patients who received dual therapy compared to triple therapy. Dual therapy was non-inferior to triple therapy concerning thromboembolic events.
AUGUSTUS/2019 [13]	4614 patients with AF and PCI	Major or CRNM bleeding	Patients planning to take a P2Y12 inhibitor to receive apixaban 5/2.5 mg 2 times a day or a VKA (target INR 2-3) and to receive aspirin (81 mg/day) or matching placebo for 6 months*	Major or CRNM bleeding was seen in 10.5% of the patients receiving apixaban, compared with 14.7% of those receiving a VKA, and in 16.1% of the patients receiving aspirin, compared with 9.0% of those receiving placebo.	P2Y12 inhibitor and apixaban, without aspirin, resulted in less bleeding and hospitalization without a significant increase in ischemic events than VKA, aspirin, or both.
ENTRUST-AF PCI/2019 [14]	1506 patients with AF requiring anticoagulation who had a PCI for stable or unstable CAD	CRNM bleeding within 12 months	Edoxaban (60 mg/day) plus a P2Y12 inhibitor for 12 months or VKA in combination with a P2Y12 inhibitor and aspirin (100 mg/day, for 1-12 months)**	Major or CRNM bleeding events were seen in 128 (17%) of 751 patients with the edoxaban regimen and 152 (20%) of 755 patients with the VKA regimen.	Edoxaban with a P2Y12 inhibitor was non-inferior for bleeding compared to the VKA-based regimen, without a substantial increase in the ischemic events.

We can assess thrombosis risk based on the CHA2DS2-VASc score. CHA2DS2 stands for congestive heart failure, hypertension, age (>65 = 1 point, >75 = 2 points), diabetes mellitus, prior stroke/transient ischemic attack (2 points). VASc stands for vascular disease (peripheral arterial disease, previous myocardial infarction, aortic atheroma), and sex category (female gender). A high CHA2DS2-VASc score, recent PCI or stroke, and history of chronic kidney disease will put the patient at a high thrombotic risk [15-16]. Patients with stable CAD, optimal PCI, and less complex coronary lesions will be at less risk of subsequent thrombotic events. In the same way, we can classify patients as high and low risk for bleeding. The HAS-BLED (hypertension, abnormal renal and liver function, stroke, bleeding, labile international normalized ratios, elderly, drugs or alcohol) score provides a practical tool to assess patients with a high bleeding risk [17]. Hence, we can divide the patients into four broad categories: (1) low thrombotic and low bleeding risk, (2) high thrombotic and low bleeding risk, (3) low thrombotic and high bleeding risk and (4) high thrombotic and high bleeding risk.

The selection between different OACs and antiplatelets is vital for patients’ outcomes. The WOEST trial concluded that clopidogrel alone plus OAC was associated with fewer bleeding episodes without significantly increasing thrombotic events than clopidogrel plus ASA and OAC. The risk of ischemia or bleeding plays a significant role in choosing between ASA and P2Y12 inhibitors. The AUGUST trial compared ASA with placebo in the patients taking the OAC and P2Y12 inhibitor. Major or clinically relevant non-major (CRNM) bleeding was seen in >12% cases receiving ASA compared to 9% in the placebo-receiving group (HR 1.89, 95% CI 1.59-2.24). There was no statistical difference in the ischemic event of placebo compared with ASA. Hence, ASA is recommended in high thrombotic and low bleeding risk patients only with double therapy (P2Y12 inhibitor and OAC).

For P2Y12 inhibitors, the main choices are clopidogrel, ticagrelor and prasugrel. For stable patients, clopidogrel is preferred to the other two for a patient already taking OAC because of the increased risk of bleeding with ticagrelor and prasugrel. For high-risk coronary patients, clopidogrel and ticagrelor both are reasonable options [18-19]. Recent clinical trials mentioned above also used clopidogrel (Table 1). Clopidogrel causes the lowest risk of bleeding, followed by ticagrelor, and so, clopidogrel should be an agent of choice from the group of P2Y12 inhibitors [20]. DOACs (apixaban, rivaroxaban, and edoxaban) have a favorable risk-benefit profile with a considerable reduction in stroke, intracranial bleeding, and mortality and comparable major bleeding as VKA, but increased gastrointestinal bleeding [21]. The AUGUSTUS trial also reported that major or CRNM bleeding was seen in 10.5% in those receiving apixaban compared with 14.7% in the group receiving VKA (HR 0.69, 95% CI 0.58-0.81). So, DOACs are preferred to VKA.

The duration of double or triple therapy should be individualized. Timing post-PCI is also an important tool to help in decision making as patients are at higher risk of thrombosis right after PCI and the risk-reducing after six months post PCI. We can divide the patients according to timeframe after PCI as well: 0-1 month, 1-6 months, 6-12 months, and >12 months.

Timeframe 0-1 month post-PCI

Patients with low thrombotic and bleeding risk, i.e., low CHA2DS2-VASc score and low HAS-BLED score, should receive ASA, P2Y12 inhibitor, and OAC for seven days, and then ASA should be discontinued. Those with high thrombotic risk and less bleeding risk, i.e., >2 score on CHA2DS2-VASc, and low HAS-BLED score should be discharged on ASA, P2Y12 inhibitor, and OAC, and ASA should be discontinued at one month post-PCI. Those with low thrombotic and high bleeding risk should be switched to a combination P2Y12 inhibitor right after PCI and should continue for at least six months. High thrombotic and high bleeding risk patients are the most challenging, and treatment options should be individualized according to each patient’s acceptable risk of bleeding. ASA can be continued for one week or up to one month post-PCI.

Timeframe 1-6 months post-PCI

During this period, all the patients should continue double therapy with the P2Y12 inhibitor and DOACs.

Timeframe 6-12 months post-PCI

Low bleeding and thrombotic risk patients and low bleeding and high thrombotic risk patients should continue double therapy for 12 months. Patients with high bleeding and low ischemic risk should stop the P2Y12 inhibitor after six months and continue only DOACs after six months. Again, for patients with high ischemic and bleeding risk, decision-making should be individualized during this period. Patients with acceptable bleeding risk should continue double therapy for 12 months post-PCI.

A recent systematic review and meta-analysis that included four trials, PIONEER AF-PCI, RE-DUAL PCI, AUGUSTUS, and ENTRUST AF-PCI (7953 patients), was published in 2020. In patients with AF and PCI, dual therapy lowers the chances of bleeding; however, its impact on death and ischemic events was still uncertain [22]. Table 2 summarizes the preferred and recommended treatment.

**Table 2:** Choice of anticoagulation with duration of PCI and bleeding risk PCI: percutaneous coronary intervention, DOAC: direct oral anticoagulant, ASA: acetylsalicylic acid (aspirin). *Risk and benefits should be discussed with patients.

Post-PCI time	Low thrombotic and low bleeding risk	Low thrombotic and high bleeding risk	High thrombotic and low bleeding risk	High thrombotic and high bleeding risk
0-1 month	DOACs + P2Y12 inhibitor	DOACs + P2Y12 inhibitor	DOACs + P2Y12 inhibitor + ASA	DOACs + P2Y12 inhibitor
>1-6 months	DOACs + P2Y12 inhibitor	DOACs + P2Y12 inhibitor	DOACs + P2Y12 inhibitor	DOACs + P2Y12 inhibitor
>6-12 months	DOACs + P2Y12 inhibitor	DOACs only*	DOACs + P2Y12 inhibitor	DOACs only*
>12 months*	DOACs only	DOACs only	DOACs only	DOACs only

Timeframe >12 months post-PCI

Two main trials, the AFIRE trial, and the OAC-ALONE trial discuss the use of double therapy or OAC alone in AF patients following 12 months post-PCI. In the AFIRE trial, rivaroxaban monotherapy was non-inferior to the combination of rivaroxaban and single antiplatelet therapy for efficacy, and superior for safety [23]. Similarly, OAC-ALONE compared the oral anticoagulant alone and combined single antiplatelet plus oral anticoagulant. This trial was unable to establish the non-inferiority of OAC independently to a combination [24]. In light of these trials, we prefer to continue only OAC, but the risk and benefits should be discussed with patients. A low bleeding and high thrombotic risk patient could benefit from prolonged combination treatment.

Lastly, treatment efficacy also depends upon compliance and adherence to the regimen. The discontinuation rate of DOACs is less than the VKA because of stable dosing, no dosage monitoring, and less drug interaction. Re-education, involvement of family members in treatment discussion, brochures, and pill organizers can improve adherence and compliance [25-26].

## Conclusions

Oral anticoagulation with a P2Y12 inhibitor plus ASA (triple therapy) carries a high risk of bleeding and death compared to OAC plus P2Y12 inhibitor (double therapy) without a significant increase in efficacy. CHA2DS2-VASc and HAS-BLED scores can be utilized to calculate the thrombotic and ischemic risk. High thrombotic and low bleeding risk patients should continue triple therapy for up to a month after PCI and then discontinue ASA. Low thrombotic and high bleeding risk patients, as well as high-risk patients for both thrombosis and bleeding, should only continue double therapy for six months post-PCI.
